# Supplementary material for: Using Artificial Intelligence to Optimize Ecological Restoration for Climate and Biodiversity
Source: Glob Chang Biol. 2026 Nov 9;32(11):e71165. doi: 10.1111/gcb.71165 (PMC13747821; doi:10.1111/gcb.71165)
Supplement: Supplementary file 1 [file GCB-32-e71165-s001.pdf]

SUPPLEMENTARY INFORMATION

Supplementary Methods and Results

Generality of carbon and biodiversity trade-offs

We tested to what extent our policies trained on a single arbitrary region could be applied to other geographic and climatic contexts, by running restoration experiments on a set of 100 additional datasets of varying geographic location, extent, and resolution (Fig. S3). Specifically, we generated 100 additional rasters of 128×128 cells with resolution at the equator of 2.5, 10, or 25 km. Their placement globally was random but conditioned on having at least 70% of cells on land (Fig. S3). For each raster we extracted elevation and climate data and simulated 500 species following the procedure described above. We then ran the five optimized policies on these datasets to evaluate the ability of our trained models to generalize outside of the region on which they were optimized.

We found that, despite such wide geographic variation and no fine-tuning to regional conditions, the policies consistently resulted in general trends reflecting the objectives used to train them. Accordingly, the net carbon gain decreased from Policy 1 to 5, the number of species with improved conservation status was higher in Policies 2–5, and costs were roughly constant across all policies (Fig. S7a–c). However, the patterns were less consistent with regards to changes in species number across risk categories (Fig. S7d–i). These results suggest that while models trained on a region can be applied with reasonable performance to other regions, they would almost certainly benefit from a region-specific re-training or fine-tuning.

Consistent with our previous experiments, we found that the restoration areas selected under different policies only partly overlap (Fig. S1b), indicating that trade-offs between carbon and biodiversity priorities are common at different geographic locations and scales. These experiments also confirmed the crucial potential of biodiversity credits in contributing to the financial feasibility of restoration policies that target both carbon and extinction risk reduction (Fig. S8a–d; Table S2).

Comparison with greedy search optimization

We carried out analyses of the simulated datasets using simple metrics to select the restoration areas instead of CAPTAIN's neural network to evaluate the performance of our trained models against simpler non-parametric approaches. We ran three models targeting carbon and cost, extinction risk and cost, or all three factors. The models used a subset of the 16 features monitored by the agent in the other experiments.

Non-parametric model (NPM) 1 focused on carbon and costs and used ratio between the potential carbon in a cell (feature 3 in our setup; see *Methods, Observed features*) and its

restoration cost (feature 16) as the priority metric to select restored areas. NPM 2 targeted carbon, extinction risk, and costs. We summarized the importance of a cell for biodiversity as the weighted average of the number of CR, EN, VU, NT, and LC species (all rescaled between 0 and 1). To maximize the coverage of protected species we only counted LC species that are not already found within previously restored cells (feature 15 in our CAPTAIN policies). The weights were proportional to those used in our CAPTAIN reward function (Eq. 10). Restoration priorities were set proportional to the ratio between the sum of carbon and biodiversity metrics and costs. Finally, NPM 3 focused on extinction risk and costs and selected the cells with highest ratio between the biodiversity metric and costs.

Through the analysis of 100 simulated datasets we found that NPM1 achieved a performance similar to that of the equivalent neural network model (Policy 1) with comparable net carbon gains and slightly lower costs (Table S3). The biodiversity outcomes of NPM2-3 (i.e. models that included extinction risk reduction in their objectives) were however generally worse than those obtained from trained policies. For instance NPM3 (which did not include a carbon objective) included on average 32 fewer species in the selected areas compared to the equivalent trained model (Policy 5). It also led to the recovery of 133 previously threatened species as opposed to the more than 200 species achieved by trained models. The restoration costs of NPM2-3 were also slightly higher than those of equivalent trained policies (Table S3). This shows that the additional computational burden of training a neural network policy is counterbalanced by an improved outcome.

Testing a larger neural network

We compared the performance of the small neural network utilized in our CAPTAIN experiments against that of a larger model. To this aim we trained additional models with two hidden layers of 16 and 8 nodes, using the same training procedure and simulated datasets (Fig. S5). These models therefore included about 7 times more parameters than the small ones.

After running the trained models on our set of 100 simulations, we found that the larger models did not yield substantially improved outcomes compared to the small models (Table S3). Costs, carbon and biodiversity metrics suggest that the richer parameterization does not lead to a significant change in performance under our simulated scenarios. These results are likely a function of the relatively small number of features used in our analyses and larger models might be beneficial in scenarios with more aspects of the environment being observed by the agent. Although the construction of the model with parameter-sharing across grid cells and rescaled features makes the number of parameters independent of the size of the area and the number of species, further exploration is needed to assess whether larger models might lead to a better performance with datasets spanning a wider extent and more complex biodiversity patterns.

Sensitivity analyses for the Atlantic Forest restoration priorities

We ran additional analyses to explore the potential impact of inherent error in the input data, focusing on the variables which we deemed most likely to influence the results: the species-specific above ground biomass (AGB; used as a proxy for carbon sequestration) and on the spatial distribution of restoration costs. While we acknowledge that these experiments do not capture all sources of uncertainty and error in the data, they serve as a demonstration of how the robustness and sensitivity of estimated restoration priorities can be assessed.

Simulating error in carbon estimates – First, we simulated error in AGB by randomly drawing for each species i, $\log(\text{AGB}^i_\varepsilon) \sim \mathcal{N}\left(\log(\text{AGB}^i), \sigma = 0.25\right)$. This resulted in a simulated error of $\approx$ 20% on the carbon associated with each species relative to our estimates. We performed 30 randomizations of the species AGB values and analyzed them under the three trained policies.

Simulating error in cost distribution – We additionally simulated error in cost distribution using Perlin noise to generate random, but spatially structured variation (Fig. S13 for an example). The random noise was centered in 1, rescaled to range from 0.75 to 1.25, and applied as a multiplier to the cost layer (Fig. S12a). This resulted in a $\pm$ 25% random variation of the costs. We performed 30 randomizations of the cost layer and analyzed them under the three trained policies.

Our analyses show that, although the prioritization maps remain broadly consistent among experiments, the additional error has a non-negligible impact on the inferred restoration priorities (Fig. S9). We measured the effect by computing the average percentage overlap between the priorities inferred from each of the 30 replicates and those identified in the absence of additional simulated error (i.e. the area shown in Fig. 4).

The average overlap across runs with error in carbon estimates was 94% for Policy 1, 79% for Policy 3, and 66% for Policy 5. The overlap decreased in the presence of spatially structured error in cost estimates, to 87% for Policy 1, 69% for Policy 3, and 51% for Policy 5. These results show that, while most of the priority areas are consistent across sensitivity tests, errors in the data can lead to more uncertain predictions, with a greater impact on restoration priorities targeting extinction risk compared to carbon (Fig. S9f, i). Our analyses show that this approach can be used to assess the sensitivity of prioritization results to data uncertainties and identify top-priority areas that are robust to error in the data, while highlighting the areas that are most prone to shifting priority.

Supplementary Tables

	β/χ			
	median	st. dev.	25%	75%
Policy 1	0	0	0	0
Policy 2	0.80	2.25	0.51	1.20
Policy 3	2.65	3.93	1.72	4.17
Policy 4	2.94	7.66	2.17	5.01
Policy 5	3.51	6.05	2.56	5.39

Table S1: Biodiversity credit valuation estimated to reach the same net financial gain as in Policy 1 (focused on carbon sequestration) across all other policies (with increasing weight associated with extinction risks), computed based on Equation 17. The values are calculated across 100 simulations based on the reference region (on which models were trained).

	β/χ			
	median	st. dev.	25%	75%
Policy 1	0	0	0	0
Policy 2	2.64	18.09	0.26	6.50
Policy 3	4.03	45.29	1.31	10.19
Policy 4	6.06	13.58	2.09	11.67
Policy 5	4.49	30.96	0.00	11.20

Table S2: Biodiversity credit valuation estimated to reach the same net financial gain as in Policy 1 across all policies, computed based on Equation 17. The values are calculated across 100 simulations based on a set of random regions differing in size and location from the region used to train the policies (Fig. S3).

	Model	Cost<br>median (q2.5, q97.5)	Sp. in restored areas<br>median (q2.5, q97.5)	Net carbon<br>median (q2.5, q97.5)
non-parametric	1	941 (591, 1260)	383 (227, 449)	846 (688, 1021)
	2	1128 (846, 1723)	431 (339, 484)	690 (528, 929)
	3	1096 (789, 1700)	452 (364, 488)	629 (469, 879)
small NN	Pol. 1	968 (665, 1225)	323 (218, 437)	838 (676, 1026)
	Pol. 2	820 (599, 1150)	479 (462, 489)	636 (495, 815)
	Pol. 3	899 (718, 1128)	483 (475, 491)	502 (391, 660)
	Pol. 4	916 (720, 1278)	482 (470, 492)	456 (337, 597)
	Pol. 5	883 (723, 1131)	484 (476, 492)	387 (307, 503)
larger NN	Pol. 1	1015 (693, 1316)	406 (235, 467)	809 (646, 1033)
	Pol. 2	817 (654, 1065)	476 (457, 488)	653 (513, 918)
	Pol. 3	833 (602, 1064)	483 (469, 491)	532 (404, 798)
	Pol. 4	885 (658, 1205)	481 (468, 491)	500 (382, 718)
	Pol. 5	924 (705, 1178)	483 (474, 492)	448 (340, 628)

	Model	Effectively protected sp.<br>median (q2.5, q97.5)	Sp. with improved status<br>median (q2.5, q97.5)
non-parametric	1	73 (32, 159)	172 (85, 317)
	2	119 (48, 231)	263 (119, 415)
	3	133 (52, 233)	295 (131, 435)
small NN	Pol. 1	62 (23, 125)	140 (62, 272)
	Pol. 2	178 (63, 247)	361 (218, 453)
	Pol. 3	204 (71, 268)	387 (250, 463)
	Pol. 4	201 (56, 274)	385 (206, 458)
	Pol. 5	201 (65, 268)	386 (239, 463)
larger NN	Pol. 1	84 (51, 145)	207 (136, 286)
	Pol. 2	165 (55, 237)	352 (197, 441)
	Pol. 3	201 (61, 259)	383 (237, 454)
	Pol. 4	205 (65, 282)	363 (203, 444)
	Pol. 5	208 (61, 279)	385 (235, 455)

Table S3: Summary of the outcomes of 100 simulations performed under different restoration models. Non-parametric models 1–3 (see *Supplementary Methods and Results*) were based on simple metrics targeting carbon and costs (1); carbon, biodiversity and costs (2); and biodiversity and costs (3). The other models (Policies 1–5) were based on CAPTAIN-trained neural networks of 3 and 2 nodes in two hidden layers (small NN) and 16 and 8 nodes (larger NN). The effectively protected species quantify the number of species that started from a threatened category (CR, EN, VU) at the beginning of the simulations but moved to a non-threatened category (NT, LC) after restoration, while the species with improved status are species that moved to a lower extinction risk. All values are given as median among simulations and 2.5 and 97.5% quantiles rounded to integer.

Supplementary Figures

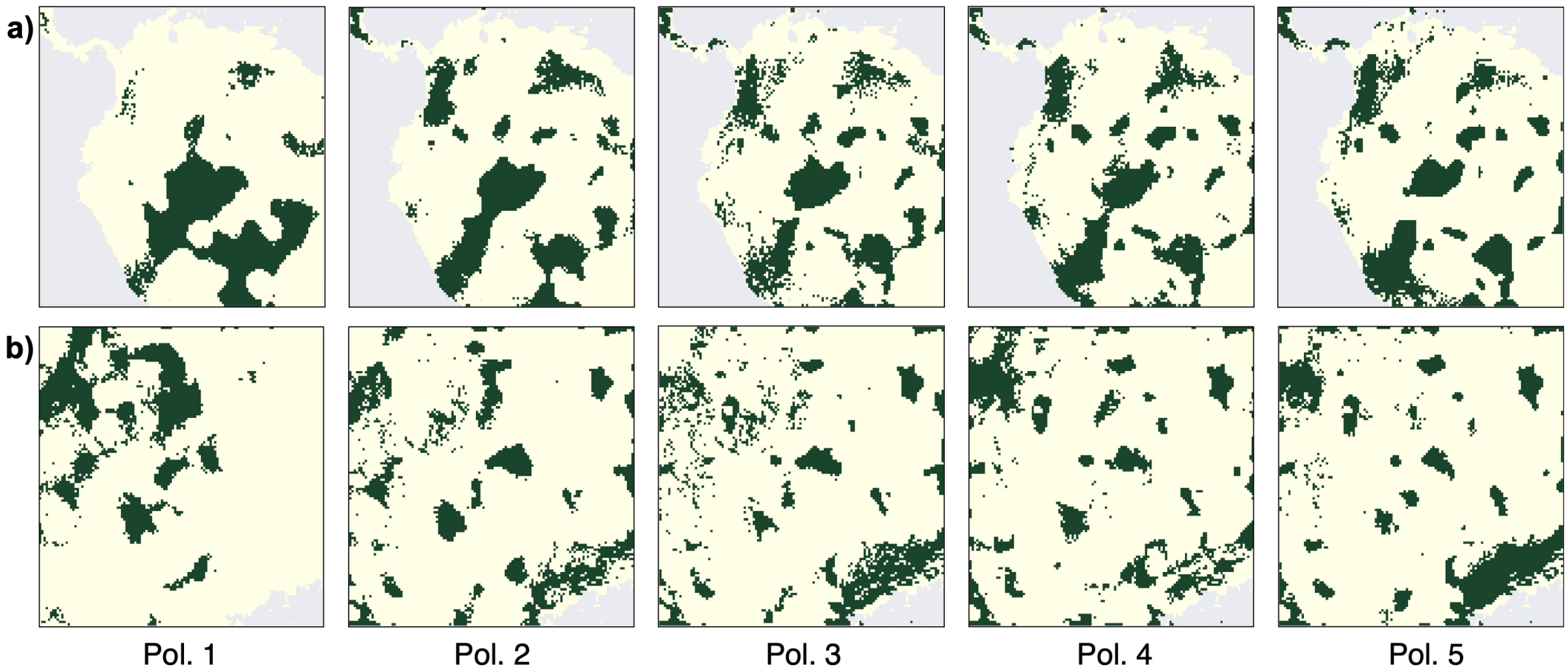

Figure S1: Changes in spatial restoration priorities depending on the relative weights of carbon (highest in Policy 1) and biodiversity (highest in Policy 5). a) Example of the restored areas in one of the 100 simulations performed on the reference region used to optimize the models (Fig. S2) selected under five policies (Table 2). Note that the placement of the restored areas depends on the specific conditions of the simulated dataset. Restored areas will therefore vary among simulations, reflecting the randomly initialized distribution of species, disturbance, and costs. b) Inferred restoration priorities obtained from simulations based on another, randomly selected region, showing similar discrepancies in spatial prioritization depending on the relative importance attributed to carbon vs biodiversity outcomes (Fig. S3).

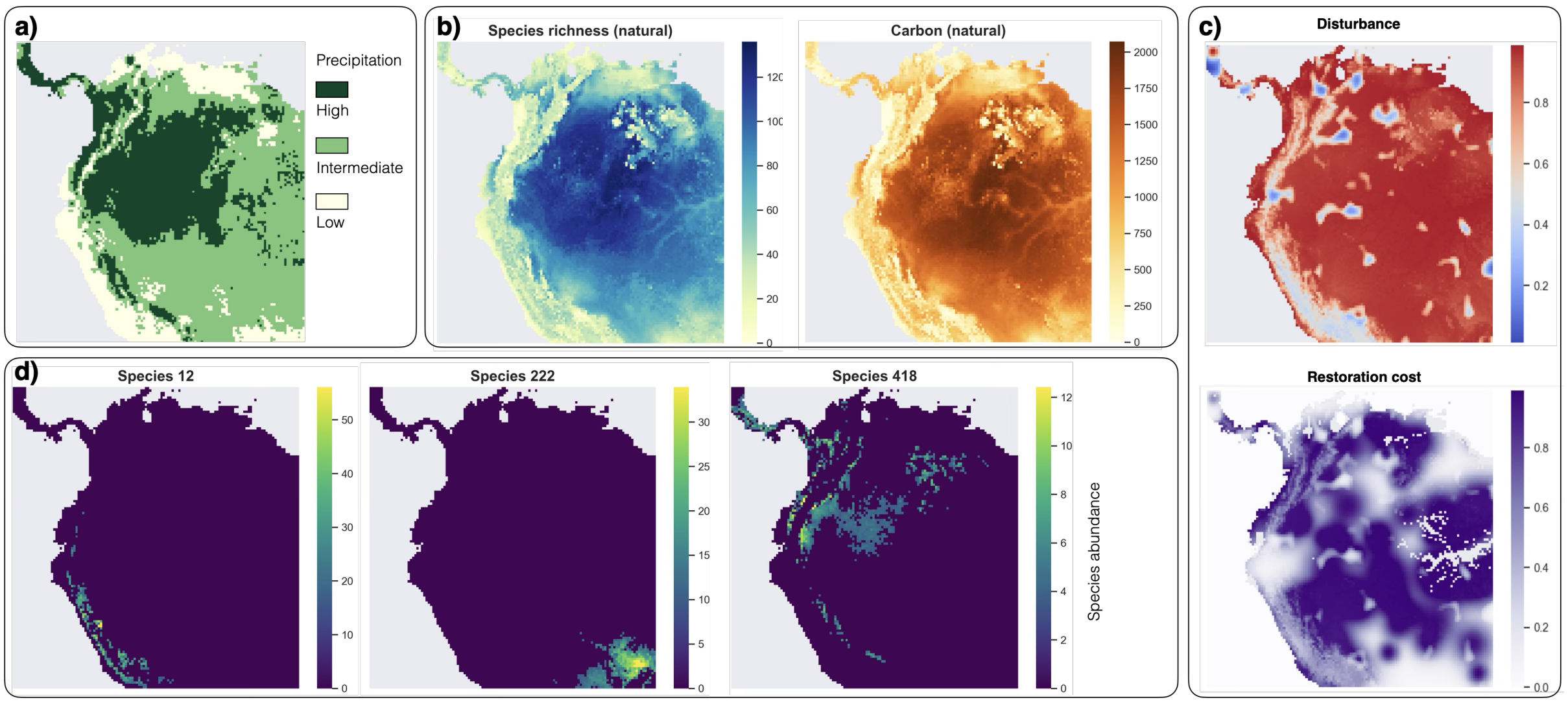

Figure S2: Map of the arbitrary area used for model training and subsequent simulations with examples of the empirical and simulated data used in our CAPTAIN runs. The physical properties of the area are based on the real topography and climate of the selected portion of Central and South America (a). The area was gridded into 128×128 cells and included empirical climate and elevation layers, while the 'natural' state of biodiversity and carbon (b) and the disturbance patterns and costs (c) were simulated and changed randomly across simulations. Species richness and carbon maps are obtained after stacking 500 simulated species (see examples in d). Note that only the map in panel a) is based on real data, while all other panels show simulated data. Similar simulations were repeated on 100 additional random maps of different size and location (Fig. S3).

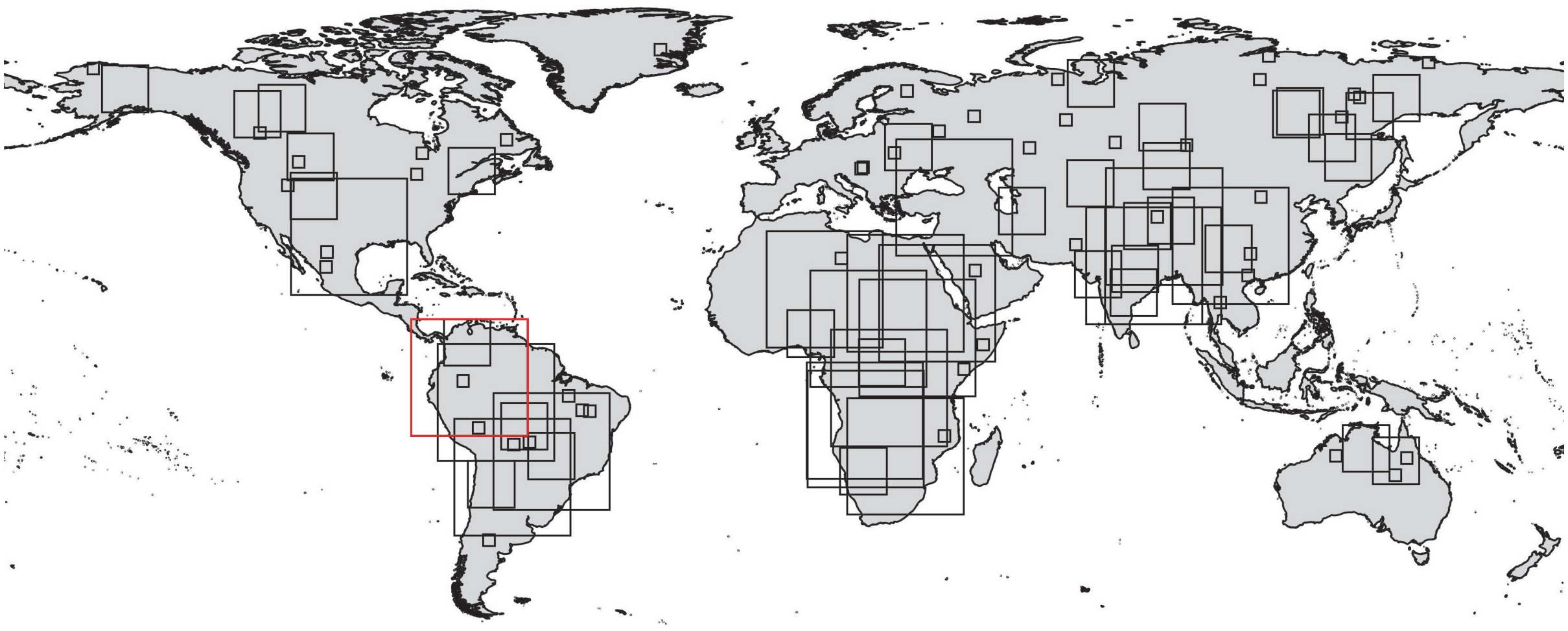

Figure S3: Maps showing the 100 randomly sampled rasters used as the basis for our simulations. The raster highlighted in red indicates the region used to train the models and to run the simulations based on a fixed geographic extent (see Fig. S2 and *Methods*).

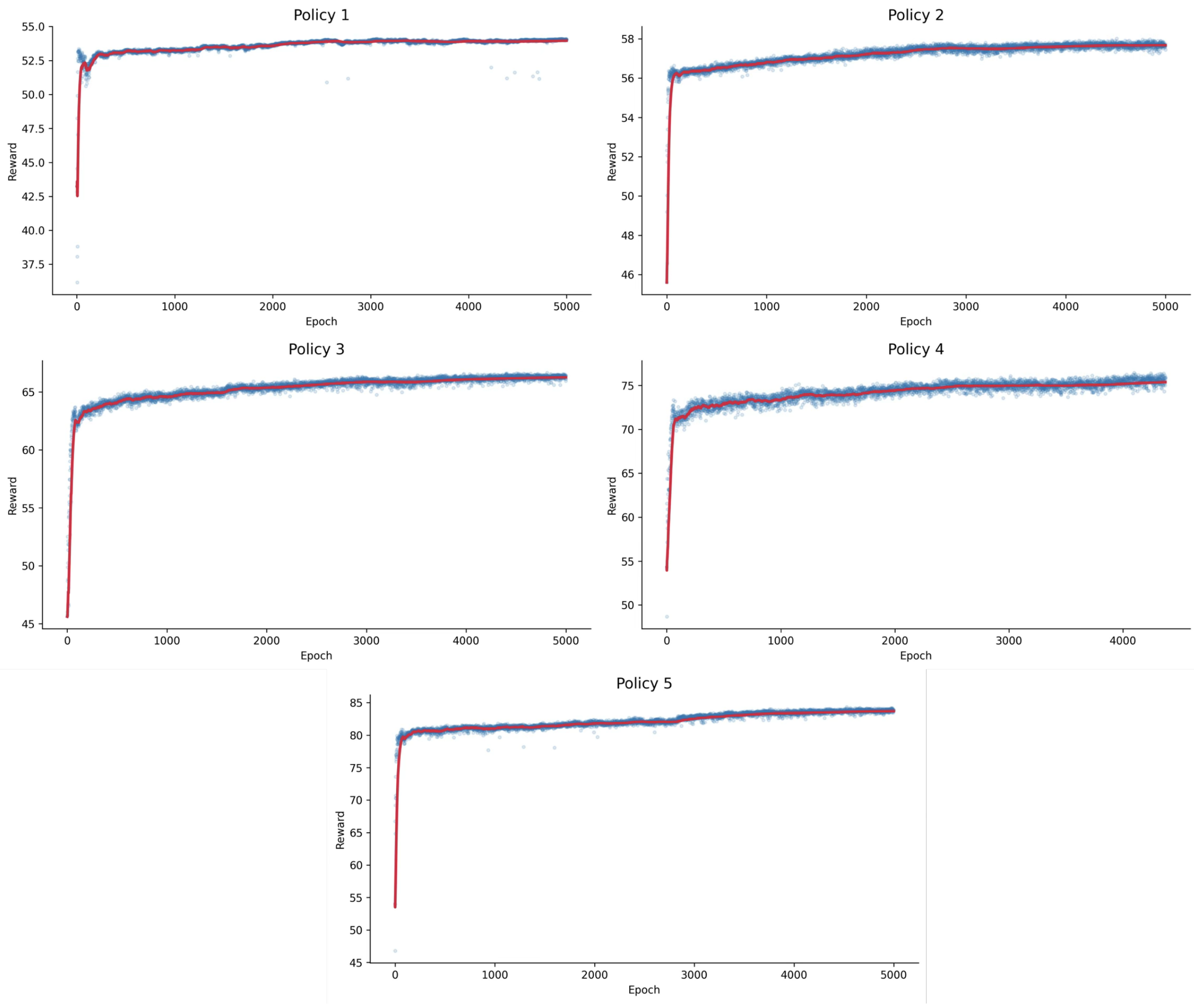

Figure S4: Training rewards across epochs based on a small policy network (see *Methods*).

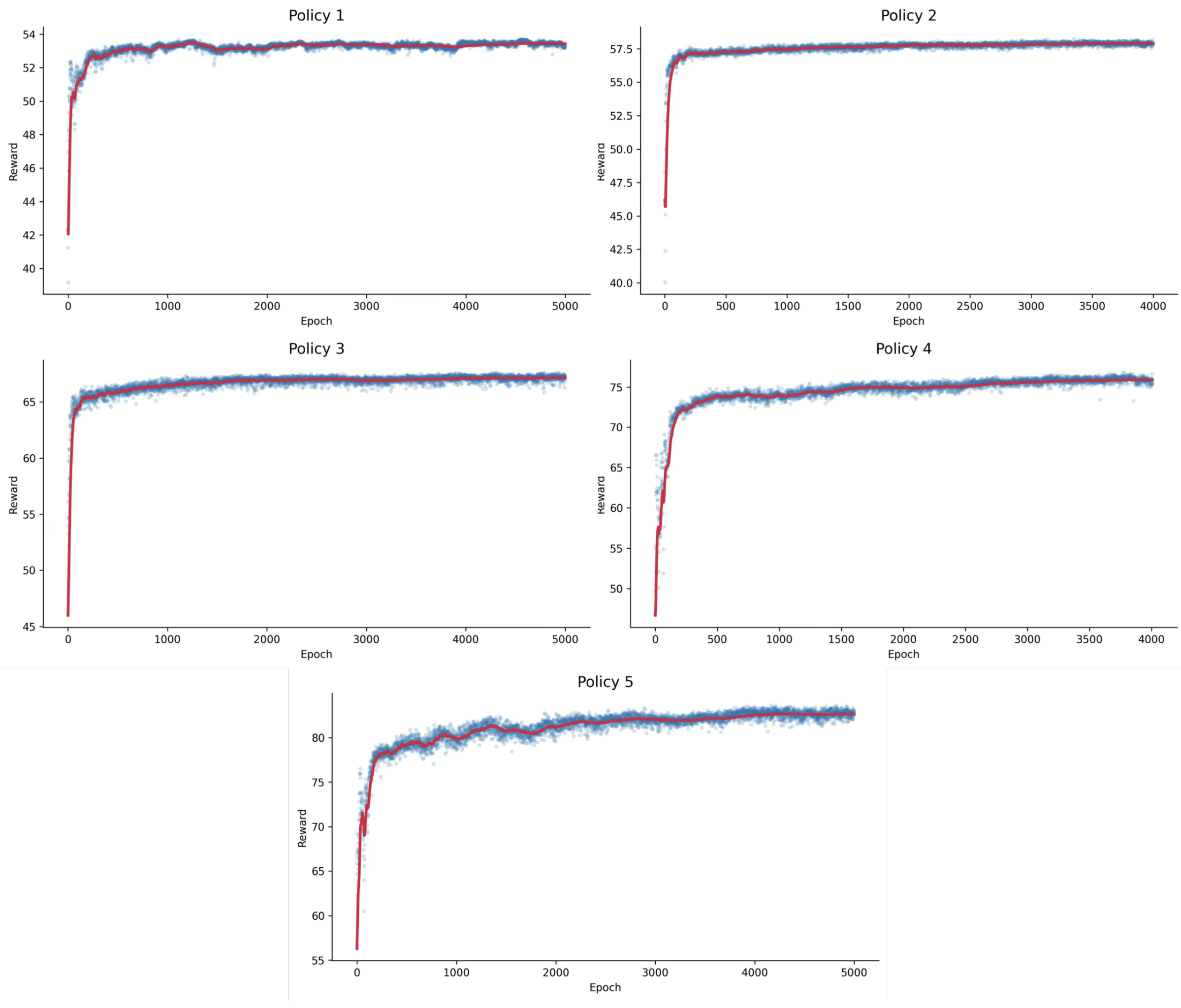

Figure S5: Training rewards across epochs based on a larger policy network (see *Methods*).

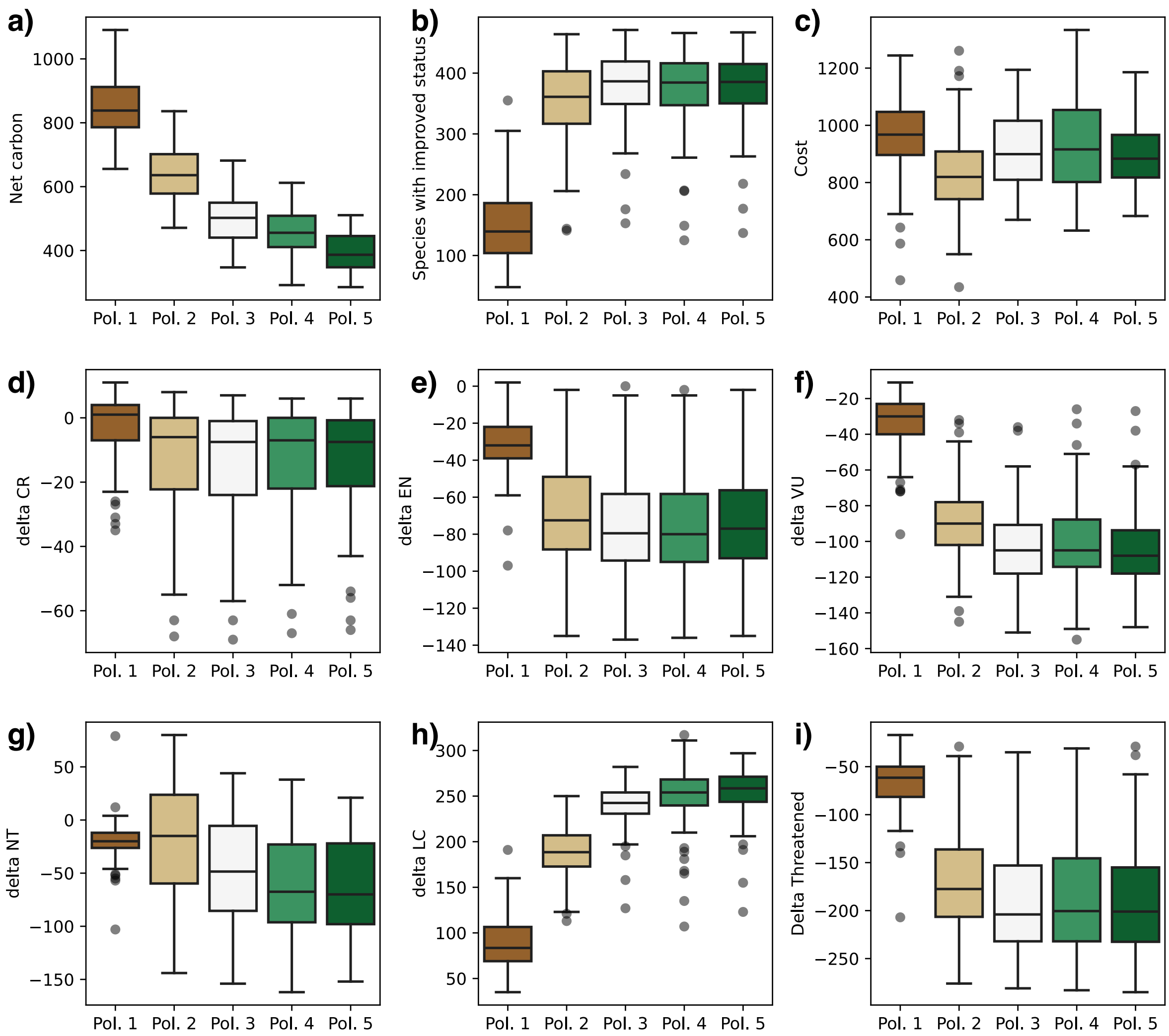

Figure S6: Summary statistics of key outcomes from 100 simulations run under five trained restoration models, Policies 1–5. The net carbon (a) represents the net gain in amount of carbon (here expressed in arbitrary units – see Methods) in the system after 30 time-steps during which 3,000 cells were restored. As the weight assigned to the carbon reward was lowered from 0.5 to 0 in favor of the biodiversity reward, the net carbon gain gradually decreased, although all restoration policies resulted in a net gain. In contrast, the number of species with improved conservation status, i.e. lower extinction risk, drastically increased as soon as a non-zero weight was assigned to the biodiversity reward (Policies 2–5; b). The implementation cost was similar across all policies (c), reflecting the fact that all models were trained with the same weight on the budget reward. Plots d–h) show the difference between the number of species in each extinction risk category, going from highest risk (CR) to lowest (LC), at the beginning of the simulation and the corresponding number at the end of the simulation. Thus, negative numbers, here observed in d–g) mean that the number of species in the corresponding risk categories decreased. Positive numbers, here mostly observed in h), mean that the number of species in these categories increased. These plots show that all policies that considered biodiversity rewards in the optimization (Policies 2–5) resulted in substantial reductions of threatened species compared to a policy targeting carbon only. This is also evident when lumping together all threatened species (i).

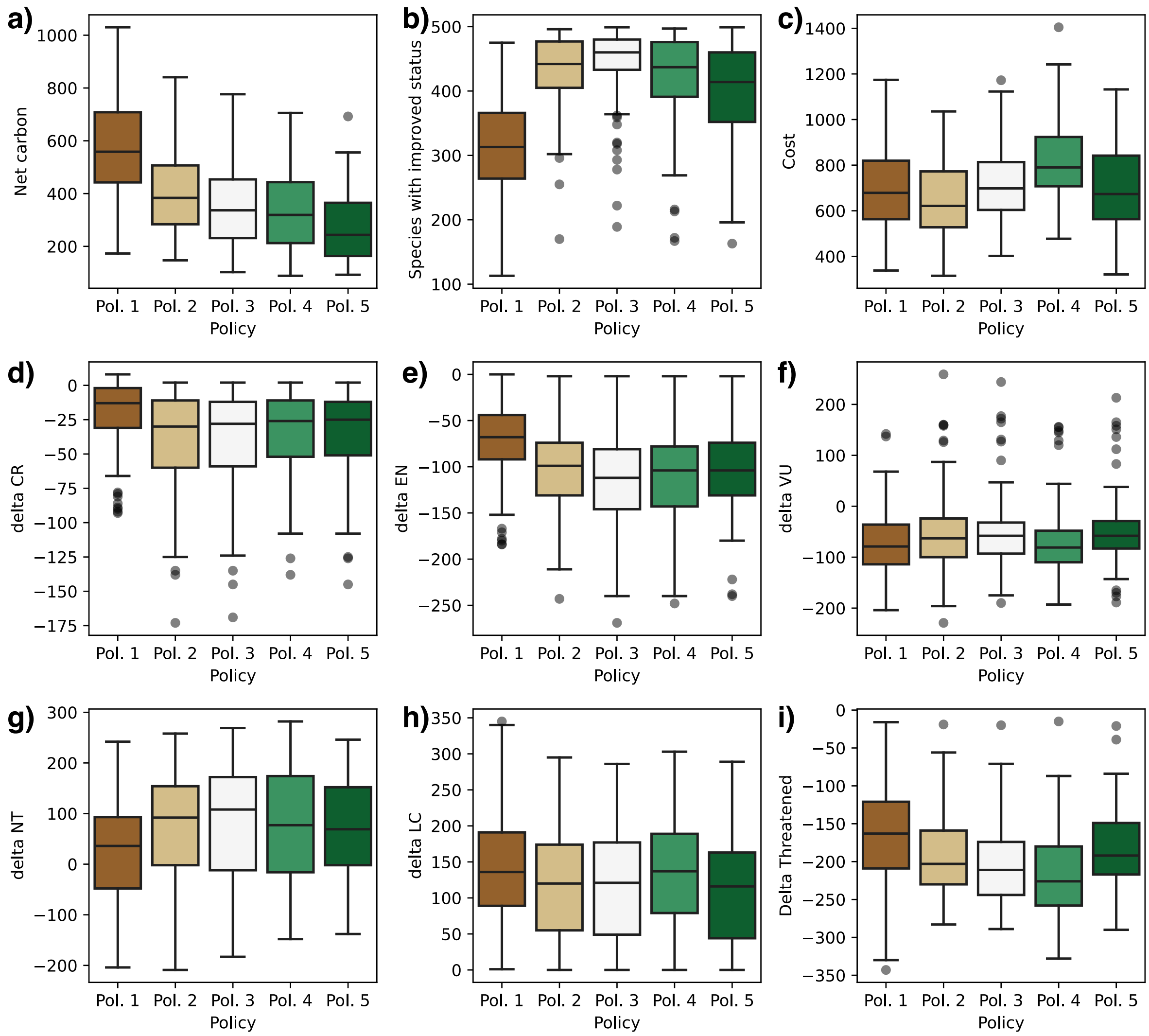

Figure S7: Summary of 100 simulations from random locations and variable resolution (Fig. S3) run under five trained restoration models, Policies 1–5. The general trends in terms of net carbon gain (decreasing from Policy 1 to 5; a), number of species with improved conservation status (higher from Policies 2–5; b), and costs (roughly constant across all policies) are similar to those observed across the simulations based on a fixed geographic extent, upon which the models were optimized. However the patterns are less consistent when looking at changes in species number across risk category (from CR to LC; d–h) or moving from a threatened to a non-threatened classification (i). These results suggest that the models (here trained on a single region) could be re-optimized or fine-tuned to best fit different regions.

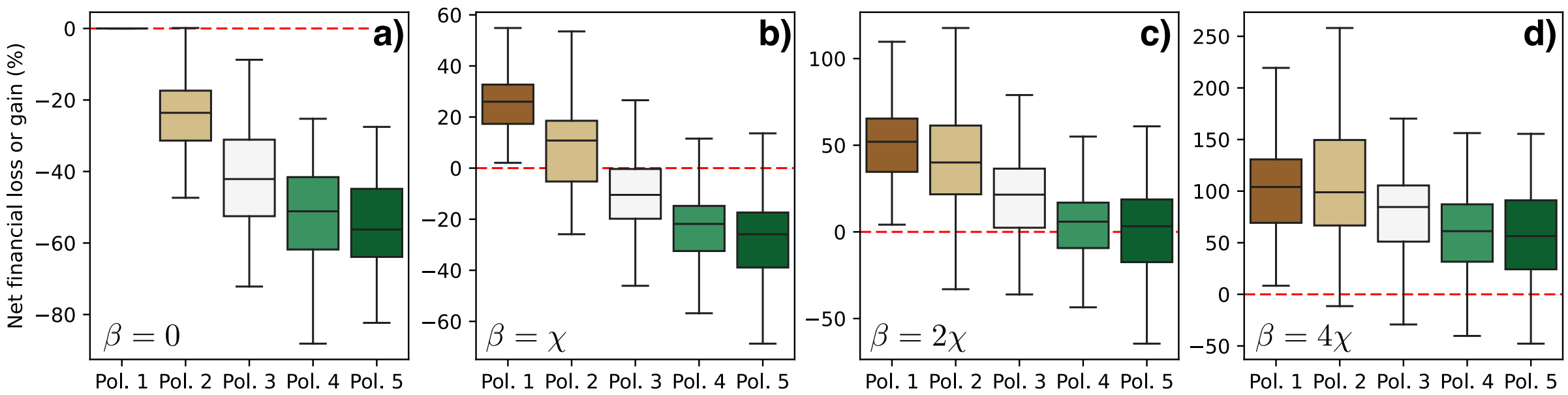

Figure S8: Financial outcomes of restoration policies (net percentage loss or gain, i.e. Φ in Equation 15) under different valuations of biodiversity credits, obtained from running the simulations across 100 random regions with different spatial location, extent and resolution. Negative values indicate a net economic loss where restoration costs exceed the cumulative value of carbon and biodiversity credits, while positive values indicate a net gain. The results are shown when only carbon credits (χ) are included (i.e. $\beta = 0$; a), which we used as a baseline of net 0 financial outcome to show how biodiversity credits could contribute value to the simulated restoration programs. The results in b–d) show that when biodiversity credits are valued at $\beta = 4\chi$, all policies become financially viable ($\Phi > 0$).

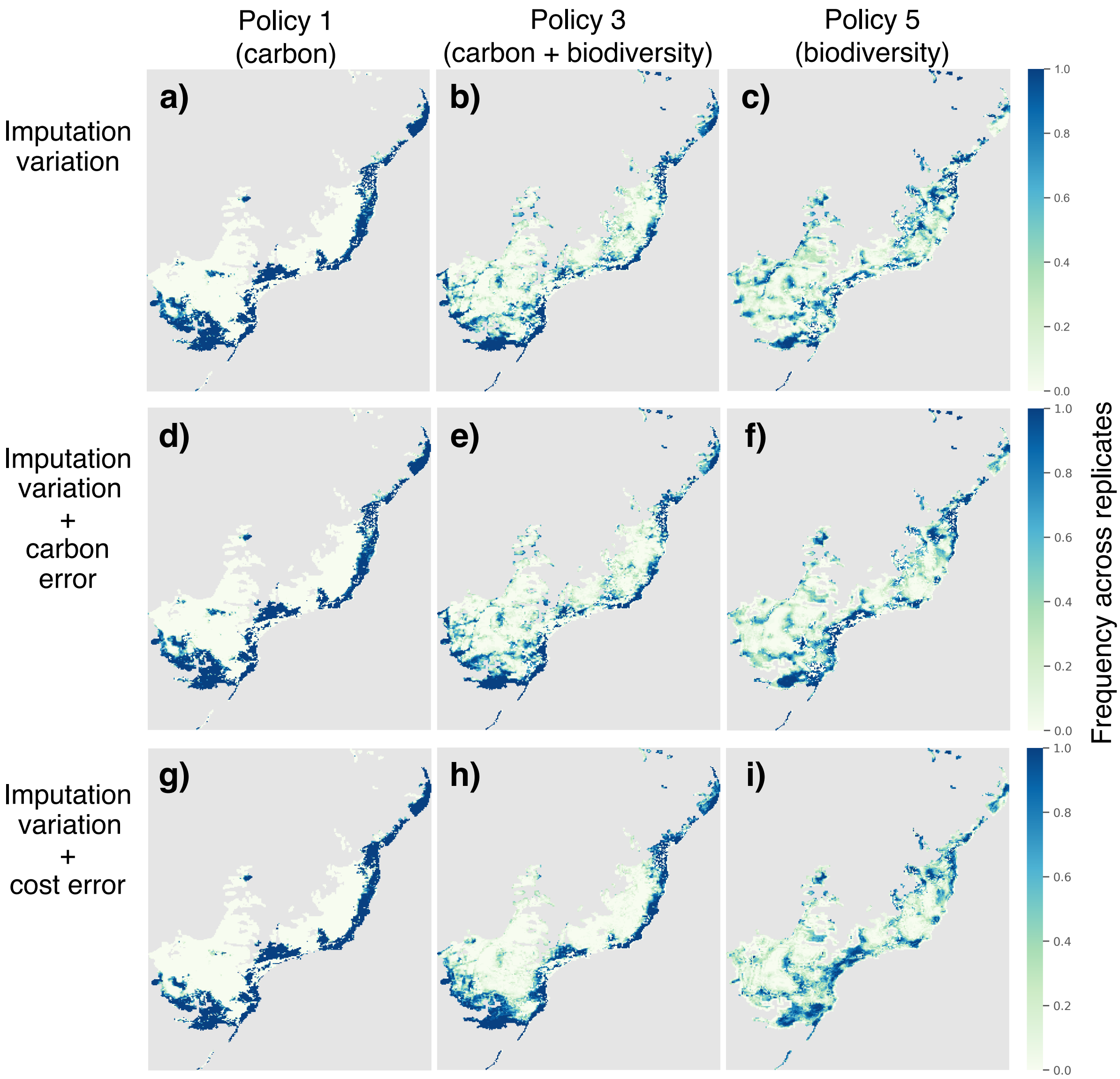

Figure S9: Restoration priorities in the Atlantic Forest: results for the three policies show the frequency at which each grid cell was selected for restoration (green-blue shades) across 30 replicates, where growth rates, species sensitivity to disturbance, and missing extinction risk assessments were randomly imputed (a–c). Sensitivity tests show the impact of simulated error in per-species carbon (d–f) and in the spatial distribution of restoration costs (g–i).

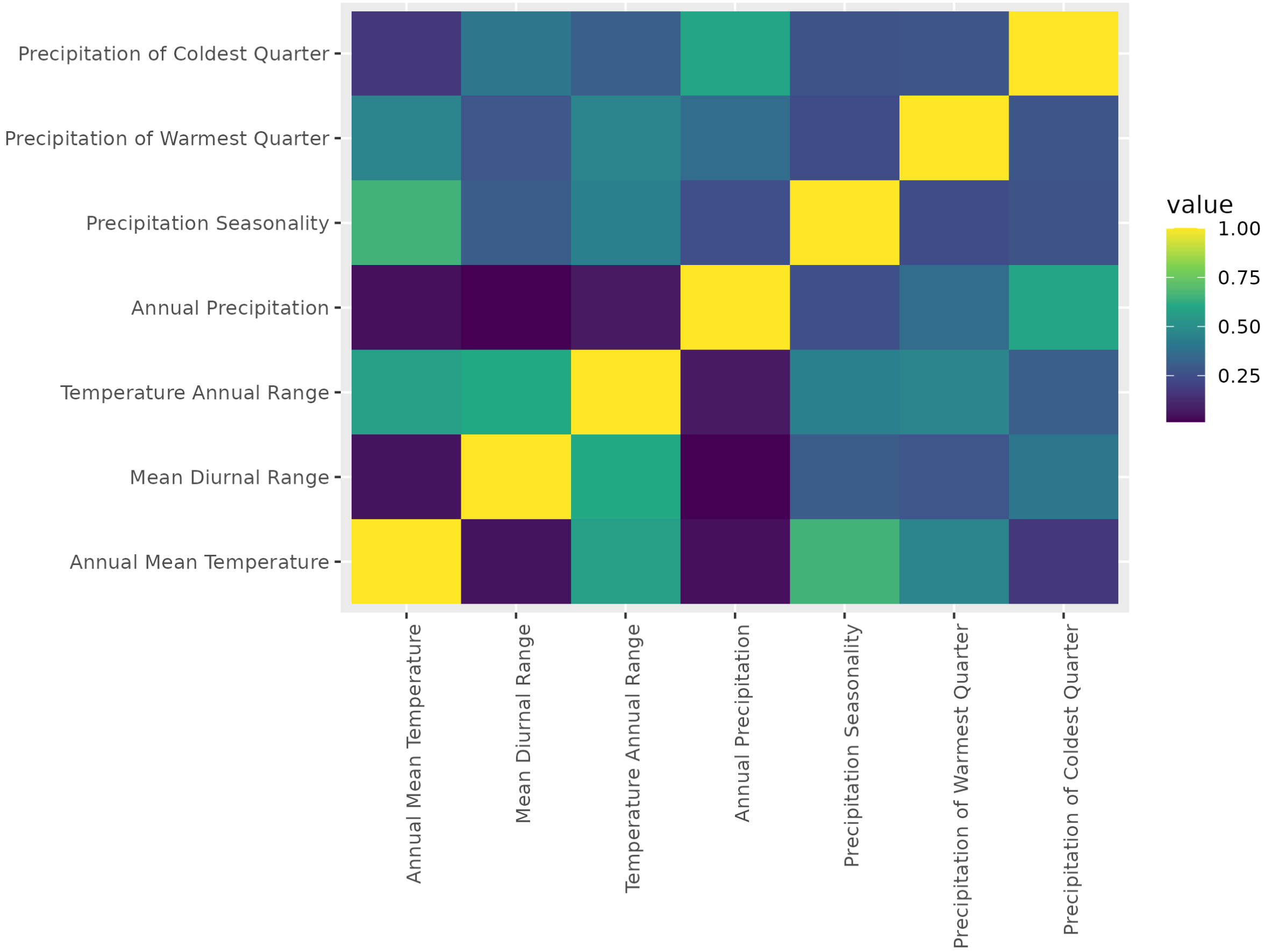

Figure S10: Correlation coefficients for the seven climatic variables[35] retained for species distribution modeling.

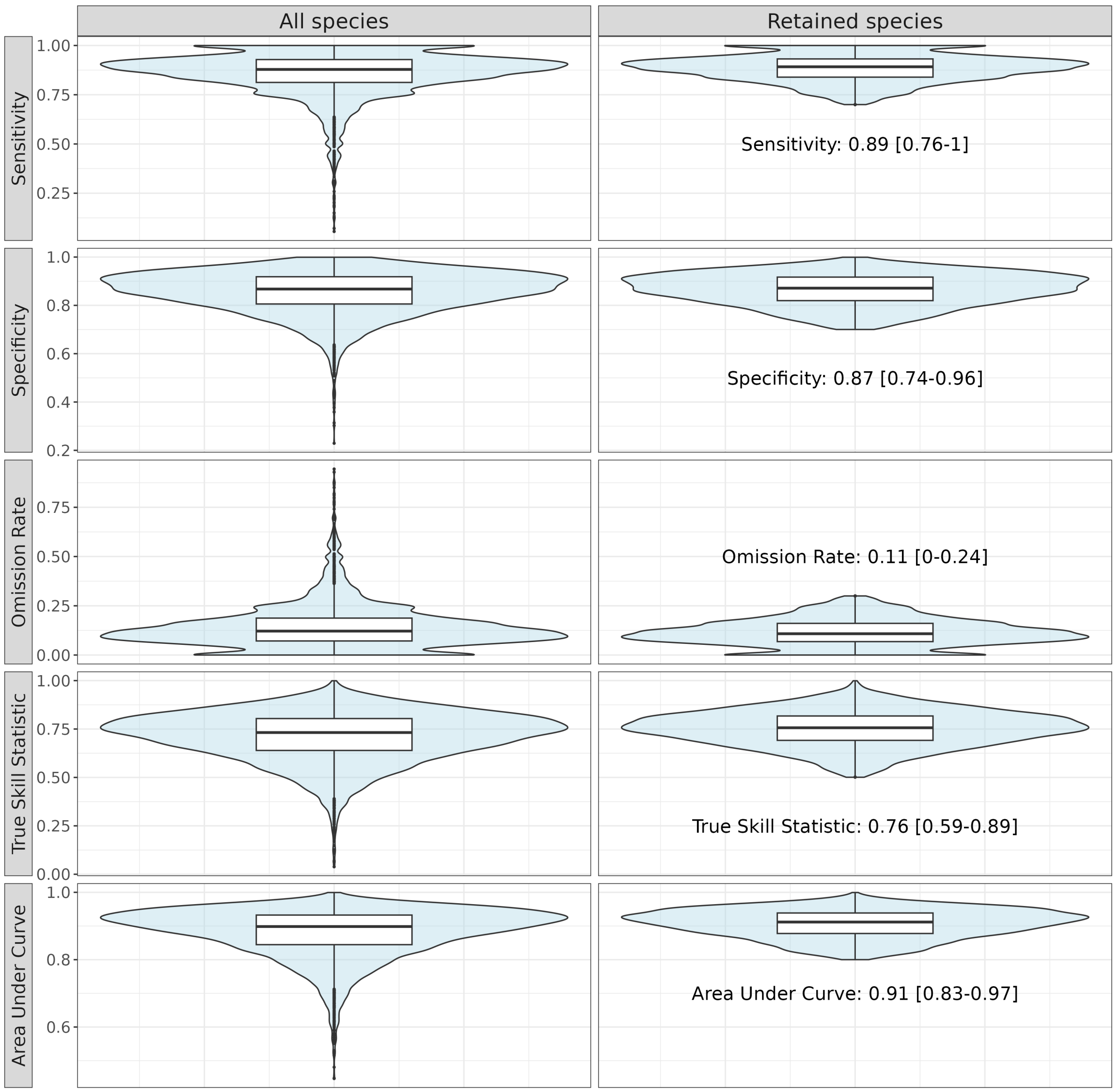

Figure S11: Violin plots and box plots of the five performance metrics of species distribution models across (a) all species and (b) retained species after applying exclusion criteria (sensitivity < 0.7, specificity < 0.7, True Skill Statistic < 0.5, and Area Under Curve < 0.8).

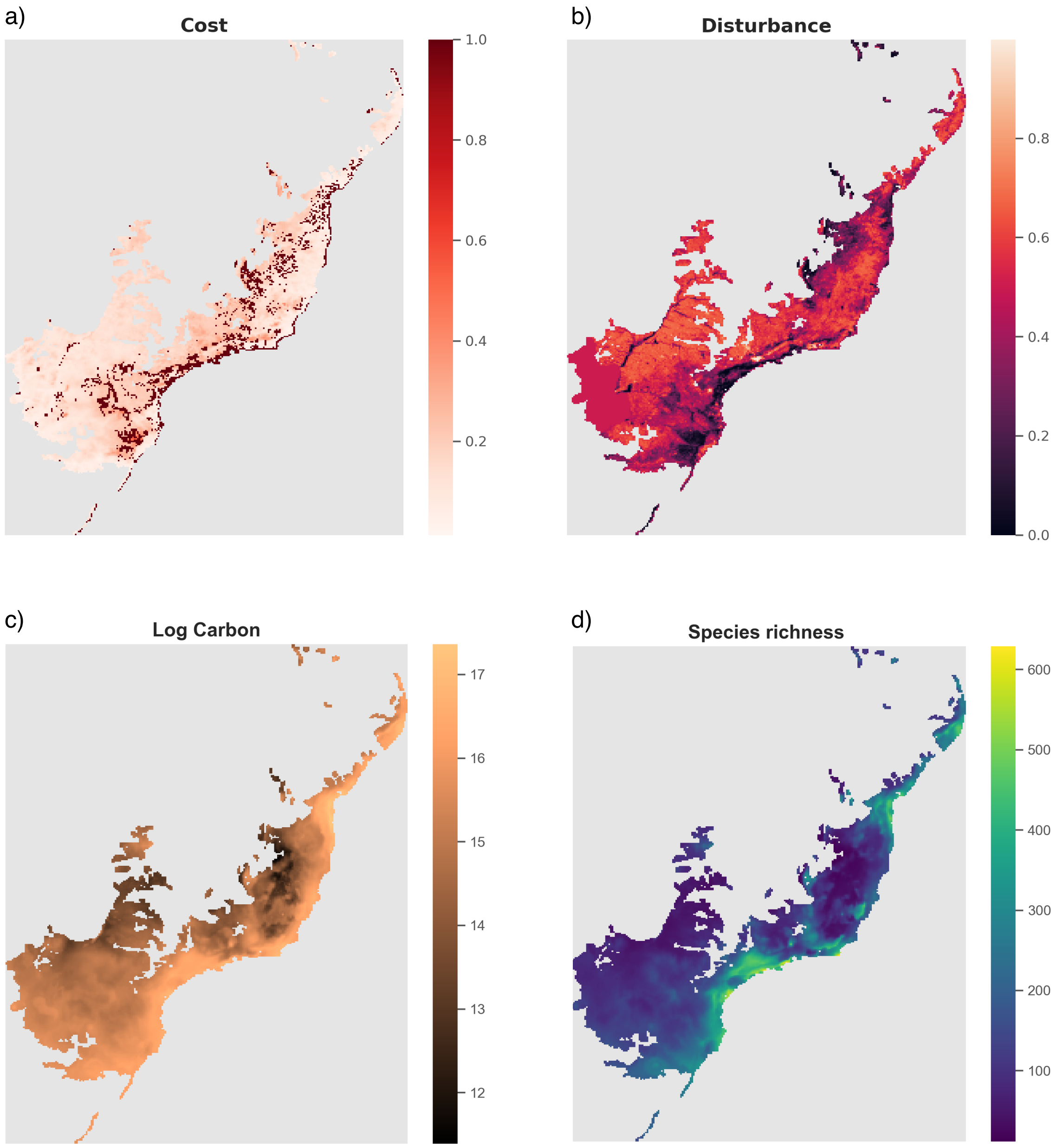

Figure S12: Approximate values of a) restoration costs, b) anthropogenic disturbance, c) simulated total carbon in the absence of disturbance, and d) simulated species richness based on stacking the species distribution models. The values are approximated as described in the *Methods*.

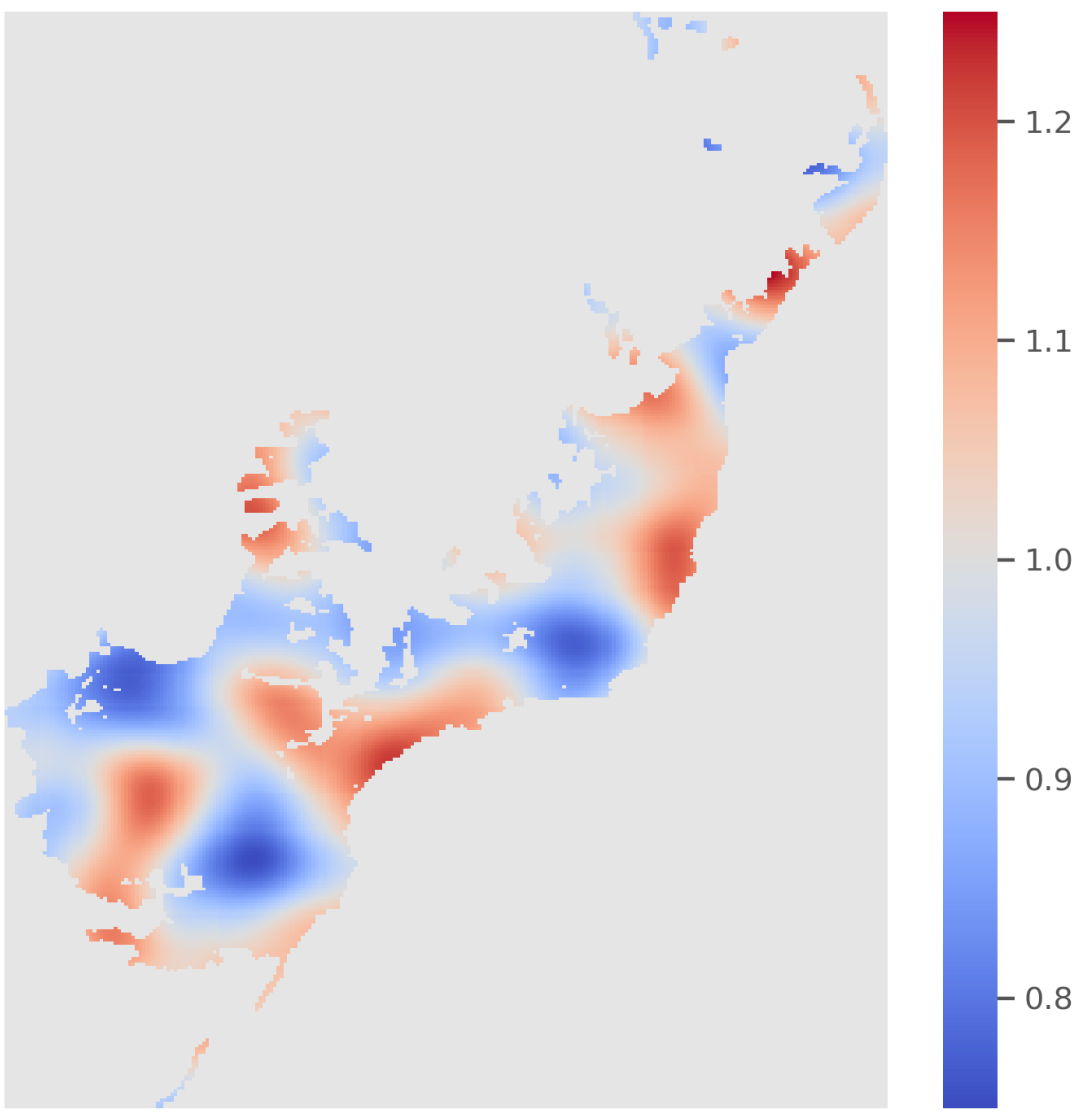

Figure S13: Example of Perlin noise applied as random cost multiplier to evaluate the impact of error in the cost layer on the inferred restoration priorities. The multiplier is rescaled to alter the cost layer by $\pm$ 25%.
